# Persistent dyslipidemia increases the longitudinal changes in telomere length

**DOI:** 10.1186/s12944-023-01938-5

**Published:** 2023-10-18

**Authors:** Xiaowei Liu, Tao Ma, Chan Yang, Juan Li, Yuhong Zhang, Yi Zhao

**Affiliations:** 1 Public Health School, Ningxia Medical University, Yinchuan, 750004 China; 2 NHC Key Laboratory of Metabolic Cardiovascular Diseases Research, Ningxia Medical University, Yinchuan, 750004 China; 3 Key Laboratory of Environmental Factors and Chronic Disease Control, Yinchuan, 750004 China; 4 School of Nursing, Ningxia Medical University, Yinchuan, 750004 China

**Keywords:** Dyslipidemia, Blood lipid levels, Changes in telomere length

## Abstract

**Background and aims:**

Leukocyte telomere length (LTL) as a ‘biological clock’ of aging is closely related to human health, its association with an aging-related disease, dyslipidemia, has been less studied and mainly focused on cross-sectional investigations.

**Methods:**

Two rounds of information and blood collections were conducted on a cohort of 1624 individuals residing in rural Ningxia, located in northwest China, with an average time gap of 9.8 years. The relative telomere length (RTL) of peripheral blood leukocytes was assessed using real-time quantitative PCR. To investigate the association between dyslipidemia, blood lipid levels, and alterations in RTL, multiple linear regression and generalized linear models were employed.

**Results:**

After conducting the follow-up analysis, it was observed that 83.3% of the participants in the study exhibited a reduction in telomere length, while 16.7% experienced an increase in telomere length. The results suggested that dyslipidemia at baseline or follow-up may increase longitudinal changes in telomere length, but it was more significant in the healthy group, especially in those aged ≥ 60 years. Furthermore, HDL-C levels in baseline and follow-up were found to be associated with longitudinal changes in telomere length, and lower HDL-C levels may be associated with increased longitudinal changes in telomere length.

**Conclusions:**

The change in telomere length is correlated with dyslipidemia and its lipid indicators especially HDL-C. Persistent dyslipidemia and a reduction in HDL-C levels may be associated with elevated longitudinal fluctuations in telomere length.

**Supplementary Information:**

The online version contains supplementary material available at 10.1186/s12944-023-01938-5.

## Introduction

Telomeres, the terminal nucleoprotein complexes located in the 3′ end of the chromosome, include telomere-binding proteins and telomeres, and they are the guanine-rich DNA repeat sequences [1]. Telomeres are similar to caps for chromosomal DNA. In the case of DNA replication, the telomeres “sacrifice” and lose parts of themselves to prevent the loss of important genetic information carried by the chromosomes. Telomeres will gradually shorten with cell division. When they shorten to a critical length, the cells will lose the abilities of division and proliferation, and finally die. Therefore, telomeres are also known as the “life clock” of cells [2]. With the continuous development of cell and molecular biology technologies, the relationship between telomere length, telomerase, telomeres and cell aging has aroused increasing attention from many scholars worldwide, and this field has become one of the hotspots in cell aging research. The length of telomeres mainly depends on two aspects, one is genetic factors, including different races, individuals and different tissues of the same individual [3, 4], and the other is environmental factors. Environmental factors such as smoking, diet, exercise and mental stress, as well as some new risk factors, including inflammation and oxidative stress, make vital effects on telomere length [5, 6].

Blood lipids are a general term of plasma neutral fats (cholesterol and triglycerides) and lipids (glycolipids, phospholipids, steroids, sterols), and they are extensively distributed in human body. Blood lipids are essential for basic living cell metabolism. Epidemiological studies have found that dyslipidemia shows a high prevalence rate globally. As estimated by the National Health and Nutrition Examination Survey data in USA from 2001 to 2016, the dyslipidemia rates in adult males and females were 63% and 51%, respectively [7], and the dyslipidemia rate in the UK was reported to be 69.9% [8]. With the economic development and social progress in China, and the changes in living habits, the blood lipid level in the Chinese population elevates gradually, and dyslipidemia rate also shows a significantly increasing trend. According to the results of one national survey in 2012, the dyslipidemia incidence rate among the Chinese adults was up to 40.40%, showing a substantial increase compared with 2002 [9]. A survey in 2019 found that the national dyslipidemia rate was 34.7%, of which the high total cholesterol (TC), high triglyceride (TG), high low-density lipoprotein cholesterol (LDL-C), and low high-density lipoprotein cholesterol (HDL-C) rates were 7.5%, 14.1%, 6.0% and 19.2%, respectively [10].

There are many studies on the telomere length of peripheral blood leukocytes both at home and abroad. It has been recognized that telomere shortening is related to a variety of age-related diseases, including dementia, diabetes, and cardiovascular disease [11, 12]. Thus, what is the relationship between blood lipids and telomeres? However, there are few foreign studies focusing on the correlation between blood lipid levels and telomere length changes. Ashley et al. found that there existed a certain relationship between HDL-C and telomere length [13, 14]. A strong negative relationship between LDL and telomere length was reported in patients with cardiovascular complications in a case-investigation study [15]. Moreover, Alison et al. discovered that shortening of telomere length in South Asian population with type 2 diabetes was inversely associated with TG and TC [16]. Domestic studies on the influential factors of telomere length mostly concentrate on cross-sectional studies or some specific diseases, and there are few studies on general healthy populations or longitudinal studies. Based on a 6-year cohort study published in the Journal of Diabetes in China, changes in DNA telomere length during this period were not significantly related to baseline blood lipids [17]. In the meanwhile, a case-control study suggested that telomere wear and tear led to an increased gestational diabetes risk among the pregnant women, and changes in blood lipid levels had a critical effect on this risk and pathogenesis [14]. The objective of this study was to evaluate alterations in telomere length and lipid levels within a longitudinal sample of the general population. Additionally, the study aimed to explore the association between longitudinal changes in telomere length of peripheral blood leukocytes and lipid levels. This investigation not only offers a theoretical foundation for the examination of clinical lipid levels and telomere length, but also provides scientific guidance for preventive strategies against telomere shortening. Furthermore, it offers insights into the mechanisms underlying telomere shortening in the aging process.

## Methods

### Study design and participants

**Fig. 1:**
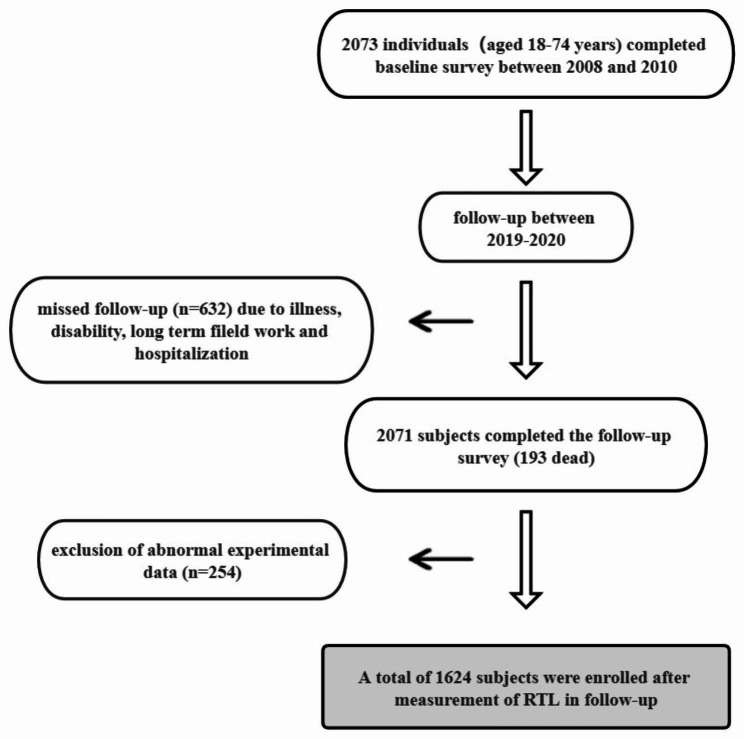
Study flow chart

The study population was from the natural population cohort of northwest (Ningxia) of “precision medicine”, a key research and development plan of the Ministry of Science and Technology. The cohort recruited participants from Pingluo County and Qingtongxia City of Ningxia Province in northwest China during the period from 2008 to 2012. By 2009, totally 2703 people had completed the baseline survey and during 2019–2020, the follow-up survey was carried out. A total of 2071 participants finished follow-up, including 1878 completing the questionnaire survey, biological sampling, and body measurement, while 193 died, with a follow-up rate of 76.62%. After excluding 254 individuals who lacked the relative telomere length data at baseline and follow-up, 1624 subjects were enrolled in this study (Fig. 1). Due to some losses to follow-up in this study, we compared baseline conditions between follow-up and lost-to-follow-up groups. Age and LDL-C levels were of great difference between the two groups, as shown in Table 1. The variables did not differ between the two groups (Supplement Table 1).

### Information collection and laboratory tests

In-person interviews were performed through well-trained scholars with the purpose of obtaining information on socioeconomic features, such as demographics (including age, gender, educational attainment, and marital status), lifestyle behaviors (including smoking, tea and alcohol drinking status, and physical exercise), as well as history of diseases. Height, weight, blood pressure (BP), hip circumference (HC) and waist circumference (WC) constitutes anthropometric measurements. Among them, height and weight were determined with light indoor clothing and with no shoes. Using an automated BP monitor, the measurement of Brachial BP was made by adopting a 5-min rest. The calculation of body mass index (BMI) was made below, BMI = weight (kg)/height (m)^2^. HC and WC were determined with multifrequency bioelectric impedance analysis (BIA, InBody370 system, XYZ) separately. After 8-hour fasting, blood samples were collected in the morning. Physicians gathered 5 ml of peripheral venous blood from the participants into a nonanticoagulant tube as well as 2 ml into an EDTA-anticoagulant tube. A One Touch Ultra2 (Life Scan, USA) was used to identify fasting plasma glucose (FPG) at baseline. In addition, the enzyme-linked immune chemiluminescence method was used to measure serum insulin at baseline. Total cholesterol (TC), triglyceride (TG), and high-density lipoprotein cholesterol (HDL-C) at baseline were identified with the enzymatic method (CHOD-PAP, Roche Diagnostics GmbH). In addition, the Friedewald formula was used to calculate LDL-C. In the following survey, biochemical auto-analyzers (Mindray BS-430, Shenzhen, China) was used to measure TC, TG, HDL-C, and LDL-C levels.

### DNA extraction and RLTL

To extract genomic DNA in peripheral blood-derived leukocytes, D3392-04 DNA Blood Mid Kit (Bao Bioengineering Co., Ltd., Japan) was utilized in this study. The Biospec-nano instrument (Shimadzu, Japan) was used to measure DNA content and quality. Then, OD260/OD280 ratio was determined within the range between 1.6 and 1.9. The real-time fluorescence quantitative PCR (Bio-Rad, Germany) was used to measure RLTL in line with the previous description by Cawthon [18]. PCR was performed in separate 96-well plates, which were divided into two parts including the telomere (T) and the housekeeping gene 36B4 (S). Each plate needs to contain the reaction of the internal reference gene and a negative control. The cycle conditions for telomere amplification included: 95℃ for 10 min, activation of FastStart Enzyme (Bao Bioengineering Co., Ltd., Japan), denaturation at 95℃ for 15 s, and annealing at 54℃ for 2 min, for totally 22 cycles. The cycling conditions for the 36B4 gene were presented: the initial conditions were the same as those for telomeres, while the annealing conditions were 58℃ for 2 min, for totally 30 cycles. At last, the calculation of relative T/S ratio, which reflected RLTL, was made using the ΔΔCt method based on the equations below: T/S = [2^Ct(telomere)^ / 2^Ct(36B4)^]^−1^ = 2^−ΔCt^, RLTL = 2^−ΔCt^ (need checking sampling) / 2^−ΔCt^ (reference gene) [19]. Detailed descriptions of sample handling and processing, as well as details regarding qPCR assay and quality control are summarized in the supplementary Table 3.

### Definition of related indicators

Alcohol consumption referred to alcohol consumption at least once weekly for ≥ 6 months. Smoking was defined as smoking more than 100 cigarettes in a year. Tea drinking indicated the consumption of one cup of tea every week for at least six months. Vigorous physical activity was set as exercising at least three times every week for at least 30 min every time. We measured relative telomere length twice, in first visit during 2008–2009 and in follow-up visit 10 years later (2018–2019). Relative telomere length was evaluated based on real-time quantitative PCR. Changes in telomere shortening = (baseline relative telomere length - follow-up relative telomere length). The participates were classified into three groups according to dyslipidemia condition between baseline and follow-up, including the persistently healthy group (HP), persistently dyslipidemia group (DL_both_), and dyslipidemia group at baseline or follow-up (DL_either_). Definition of dyslipidemia: total cholesterol ≥ 6.22 mmol/L, triglyceride ≥ 2.26 mmol/L, high-density lipoprotein cholesterol < 1.04 mmol/L, low-density lipoprotein cholesterol ≥ 4.14 mmol/L. Dyslipidemia is defined as a condition in which one of the above indicators is met, or if dyslipidemia has been diagnosed in the past [20].

### Statistical analysis

Continuous data were shown to be mean ± standard deviation (± S) and analyzed through chi-square test, while categorical data as frequency and percentage [N (%)] and analyzed by one-way analysis of variance (ANOVA). Pearson’s chi-square test was used in analyzing the independence of observations of two categorical data. We adopted multiple linear regression and generalized linear models for exploring relation of serum lipid-related index with telomere length. SPSS 24.0 was utilized for statistical analysis, and α < 0.05 stood for statistical significance. Relationship between the levels of blood lipid-related indexes and telomere length was discussed with a multi-linear regression and broad modelling method.

## Results

### Basic information between baseline and follow-up

During this time, a total of 2703 subjects were followed up in this cohort, and 2071 subjects were eventually followed up, with a follow-up rate of 76.62%. Excluding those who died and those with no relative telomere length, 1624 subjects were finally included in this study, as shown in Fig. 1. Table 1 gives the descriptive characteristics of the study population at both time points studied. This study cohort (n = 1624) included 970 females (59.73%) and 654 males (40.27%). The population was divided into three groups based on dyslipidemia, namely, the healthy group, the dyslipidemia group and the other group. According to the survey, except for age and SBP at baseline and DBP at follow-up, the remaining clinical variables between the three groups were significantly different. Smoking status and alcohol consumption status at baseline and follow-up were not significantly different among the three groups.

**Table 1:** Clinical characteristics between baseline and follow-up

	Total population (n = 1624)	Healthy persistently (n = 664)	Dyslipidemia in baseline or follow-up (n = 748)	Dyslipidemia persistently (n = 212)	*P-value*
ΔRTL	1.50 ± 2.98	1.25 ± 3.32	1.62 ± 2.67	1.88 ± 2.87	**0.010**
RTL in baseline	1.95 ± 2.50	1.87 ± 2.39	1.95 ± 2.51	2.19 ± 2.80	0.274
RTL in follow-up	0.44 ± 1.63	0.61 ± 2.34	0.33 ± 0.89	0.30 ± 0.61	**0.002**
Gender (male)	654(40.3%)	286(43.1%)	278(37.2%)	90(42.5%)	0.061
**Baseline**					
Age (years)	47.31 ± 10.56	46.82 ± 11.13	47.55 ± 10.22	47.99 ± 9.82	0.261
BMI(kg/m^2^)	23.57 ± 3.19	23.08 ± 3.15	23.66 ± 3.16	24.68 ± 3.09	**< 0.001**
WC (cm)	80.84 ± 9.35	79.52 ± 9.35	81.42 ± 9.29	82.94 ± 9.63	**0.044**
HC (cm)	92.28 ± 5.99	91.75 ± 5.84	92.32 ± 5.98	93.80 ± 6.23	**< 0.001**
SBP (mmHg)	123.80 ± 19.09	123.34 ± 19.07	123.66 ± 18.58	125.73 ± 20.86	0.276
DBP (mmHg)	79.15 ± 11.30	78.16 ± 11.26	79.30 ± 10.89	81.74 ± 12.44	**< 0.001**
TC (mmol/L)	3.93 ± 0.76	3.85 ± 0.60	3.98 ± 0.71	4.00 ± 1.20	**0.002**
TG (mmol/L)	1.33 ± 0.92	1.15 ± 0.40	1.32 ± 1.06	1.94 ± 1.23	**< 0.001**
HDL-C (mmol/L)	1.30 ± 0.30	1.41 ± 0.25	1.29 ± 0.26	1.00 ± 0.38	**< 0.001**
LDL-C (mmol/L)	2.03 ± 0.62	1.93 ± 0.49	2.09 ± 0.56	2.16 ± 1.01	**< 0.001**
FBG (mmol/L)	5.39 ± 1.58	5.16 ± 1.77	5.46 ± 1.47	5.81 ± 1.23	**< 0.001**
Smoking (yes)	280(17.2%)	111(16.7%)	121(16.2%)	48(22.6%)	0.080
Alcohol drinking (yes)	145(8.9%)	66(9.9%)	59(7.9%)	20(9.4%)	0.387
**Follow-up**					
Age (years)	57.72 ± 10.21	56.51 ± 10.39	58.33 ± 10.12	59.37 ± 9.55	**< 0.001**
BMI(kg/m^2^)	25.32 ± 5.39	24.64 ± 3.55	25.63 ± 6.90	26.37 ± 3.51	**< 0.001**
WC (cm)	87.60 ± 9.91	86.60 ± 9.68	87.53 ± 9.73	90.95 ± 10.59	**< 0.001**
HC (cm)	94.92 ± 7.38	94.17 ± 5.40	95.14 ± 9.12	96.48 ± 5.30	**< 0.001**
SBP (mmHg)	136.28 ± 20.03	136.94 ± 20.28	134.88 ± 19.13	139.19 ± 21.90	**0.012**
DBP (mmHg)	85.65 ± 13.70	86.20 ± 13.32	84.92 ± 13.30	86.48 ± 16.01	0.140
TC (mmol/L)	4.91 ± 0.99	4.57 ± 0.77	5.14 ± 1.00	5.13 ± 1.21	**< 0.001**
TG (mmol/L)	1.70 ± 1.11	1.20 ± 0.45	1.92 ± 1.16	2.51 ± 1.57	**< 0.001**
HDL-C (mmol/L)	1.75 ± 0.51	1.62 ± 0.38	1.87 ± 0.60	1.80 ± 0.45	**< 0.001**
LDL-C (mmol/L)	3.83 ± 1.67	2.75 ± 0.59	4.57 ± 1.79	4.62 ± 1.68	**< 0.001**
FBG (mmol/L)	5.81 ± 1.93	5.50 ± 1.28	5.94 ± 2.22	6.32 ± 2.33	**< 0.001**
Smoking (yes)	213(13.1%)	92(13.9%)	91(12.2%)	30(14.2%)	0.574
Alcohol drinking (yes)	212(13.1%)	91(13.7%)	96(12.8%)	25(11.8%)	0.749

### Change in relative telomere length between the three groups

**Fig. 2:**
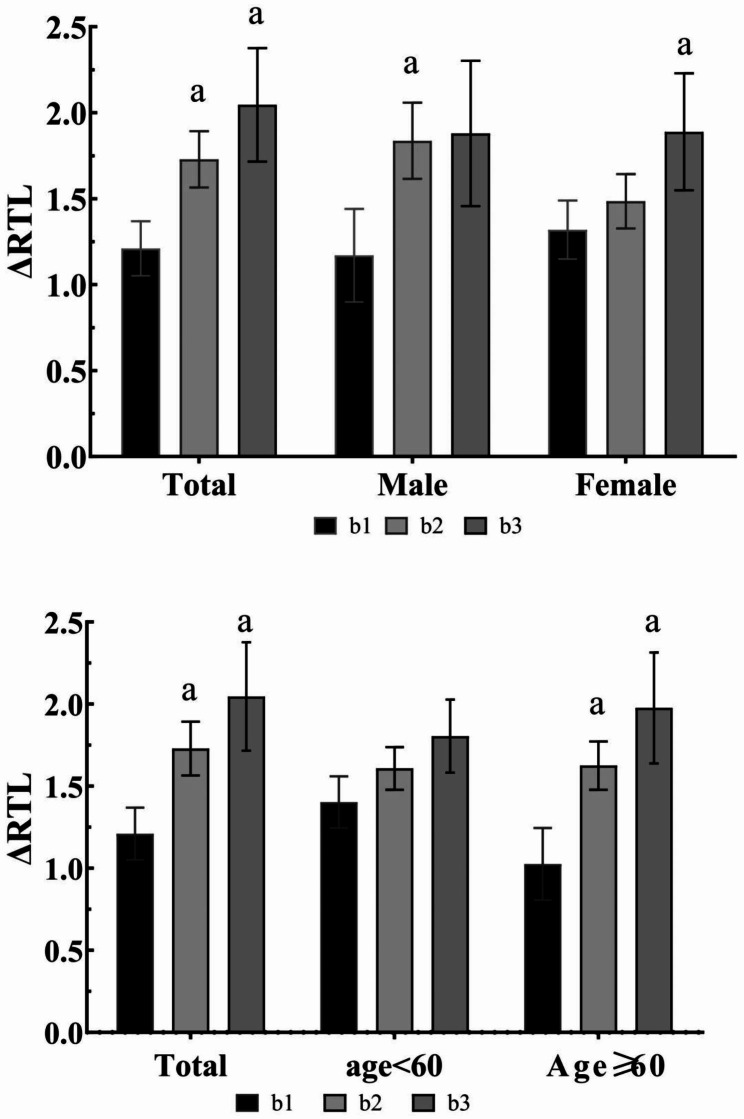
Changes in dyslipidemia status and telomere length from baseline to follow-up Note: a Compared with the continuous blood lipid healthy group, P < 0.05, and the Dunnett test was used for pairwise comparison; b1 means persistently healthy, b2 means dyslipidemia at baseline or follow-up, b3 means persistently dyslipidemia

Figure 2 shows the change in relative telomere length in the three groups. There was a statistically significant difference between the persistently dyslipidemic group and the persistently dyslipidemic group at baseline or follow-up compared to the persistently healthy group, with an increasing trend and the greatest change in telomere length in the persistently dyslipidemic group, with the same finding in the group older than 60 years. The second group among males and the third group among females were significantly different when compared with the persistently healthy group using the Dunnett test.

### The relationship between change in relative telomere length and different groups

We used a generalized linear model to discuss the relationship between the change in relative telomere length and the dyslipidemia subgroup. As shown in Table 2, using the healthy persistently group as a reference, dyslipidemia in the baseline or follow-up group and the dyslipidemia persistently group showed a positive correlation with the change in telomere length, i.e. B-values greater than 0, and the dyslipidemia persistently group had greater B-values than dyslipidemia in the baseline or follow-up group, and it was found that the change in relative telomere length was greater in the dyslipidemia persistently group, a phenomenon consistent with the results in people aged > 60 years. However, comparing the sexes separately we found different results for males and females, with the other groups showing a positive correlation with telomere length in males and the dyslipidemia group showing a positive correlation with telomere length in females.

**Table 2:** Generalized linear model between dyslipidemia status and change in telomere length

	Healthy persistently		Dyslipidemia in baseline or follow-up		Dyslipidemia persistently	
	B (95%CI)	*P*-value	B (95%CI)	*P*-value	B (95%CI)	*P*-value
Total	1 (Reference)		0.361(0.05–0.67)	**0.023**	0.630(0.17–1.09)	**< 0.001**
Male	1 (Reference)		0.667(0.12–1.21)	**0.017**	0.710(-0.08-1.50)	0.077
Female	1 (Reference)		0.166(-0.20-0.53)	0.376	0.570(0.02–1.12)	**0.044**
< 60	1 (Reference)		0.206(-0.19-0.60)	0.303	0.402(-0.19-1.00)	0.184
≥ 60	1 (Reference)		0.600(0.09–1.11)	**0.020**	0.951(0.23–1.68)	**0.010**

### Association between lipid indicators and changes in telomere length

**Fig. 3:**
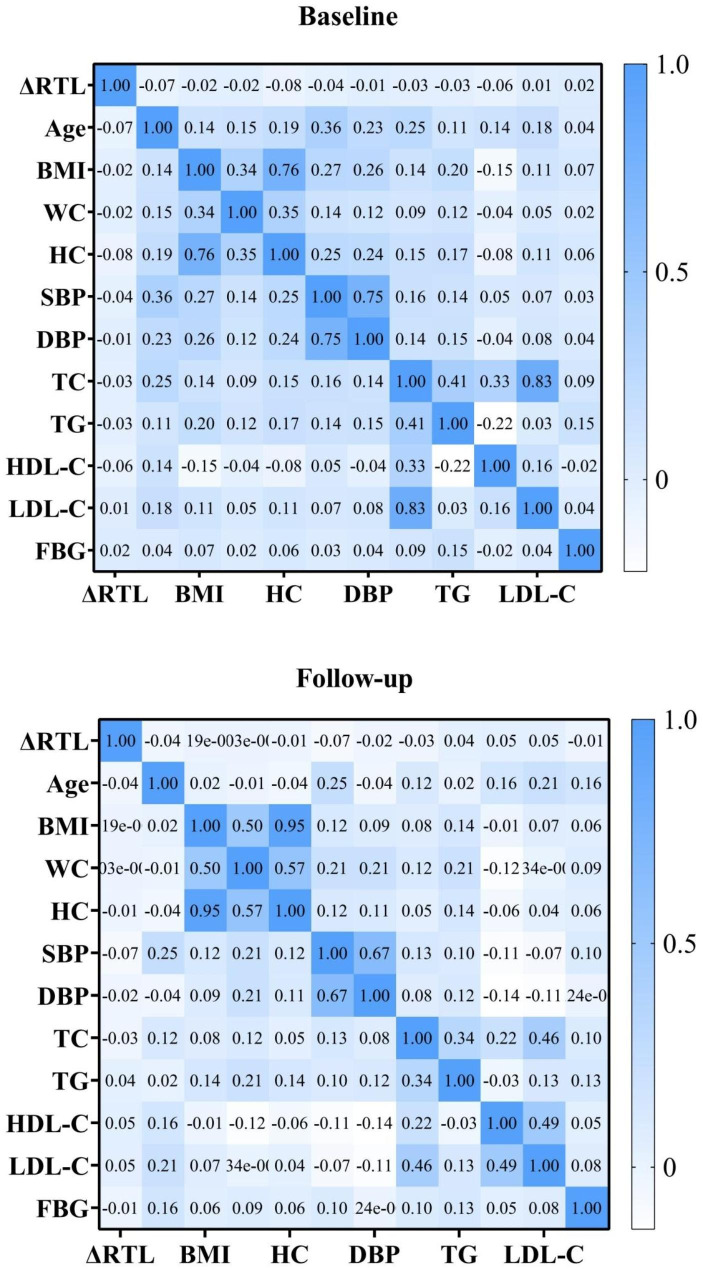
Correlation plot between change in relative telomere length and clinical characteristics at baseline and follow-up

Figure 3 and supplement Table 2 describe the association of telomere length changes and clinical variables, respectively, and we can see that HDL-C at baseline and follow-up had negative associations with changes in telomere length. The results showed that HDL-C negatively influenced the change in telomere length, i.e., the lower the HDL-C level was, the greater the change in telomere length.

In the stepwise regression analysis, a multiple linear regression model with the dependent variable Y and the independent variable X is tested for the model and each independent variable. When the model is not significant, the linear relationship of the regression model is not valid; if any of the independent variables in the model is not significant for the dependent variable, it is eliminated, and if the independent variable is significant for the dependent variable, it is filtered out and the multiple linear regression model is rebuilt without the independent variable, so as to obtain the optimal regression model [21]. As seen in the baseline multiple linear regression (Table 3), excluding the effect of multicollinearity, the results showed that the regression equation was significant, F = 2.674, p < 0.05. Of these, HC (β=-0.074, P = 0.000) was a significant negative predictor of change in telomere length, BMI (β = 0.080, P = 0.048) was a significant positive predictor of change in telomere length, while other variables did not predict telomere length change (P > 0.05), and together, these variables explained 14.3% of the variance in change in telomere length. At the same time, multiple linear regression of the change in telomere length was performed in follow-up, excluding the effect of multicollinearity and the results showed that the regression equation was significant, F = 2.614, p < 0.05. Of these, SBP (β=-0.012, P = 0.028), HC (β=-0.074, P = 0.031) and TC (β=-0.223, P = 0.013) were significant negative predictors of telomere length change, TG (β = 0.155, P = 0.034) was a significant positive predictor of telomere length change, and other variables did not predict telomere length change (P > 0.05), together, these variables explained 13.2% of the variance in telomere length change (Table 3).

**Table 3:** Stepwise multiple linear regression analysis of changes in telomere length (baseline and follow-up)

		B	P	F	R square
Baseline	Age (years)	-0.015	0.087	2.674*	0.143
	BMI (kg/m2)	0.080	**0.048**		
	WC (cm)	0.002	0.577		
	HC (cm)	-0.074	**0.000**		
	SBP (mmHg)	-0.006	0.362		
	DBP (mmHg)	0.010	0.363		
	TC (mmol/L)	-0.129	0.735		
	TG (mmol/L)	-0.051	0.757		
	HDL-C (mmol/L)	-0.438	0.271		
	LDL-C (mmol/L)	0.297	0.434		
	FBG(mmol/L)	0.057	0.270		
Follow-up	Age (years)	-0.011	0.188	2.614*	0.132
	BMI (kg/m2)	0.086	0.054		
	WC (cm)	0.012	0.221		
	HC (cm)	-0.074	**0.031**		
	SBP (mmHg)	-0.012	**0.028**		
	DBP (mmHg)	0.008	0.309		
	TC (mmol/L)	-0.223	**0.013**		
	TG (mmol/L)	0.155	**0.034**		
	HDL-C (mmol/L)	0.257	0.126		
	LDL-C (mmol/L)	0.093	0.099		
	FBG(mmol/L)	-0.011	0.779		

## Discussion

This is the prospective population-based observational study. It describes the effect of dyslipidemia on longitudinal alterations of telomere length and examines how various indicators of lipids affects longitudinal changes in telomere length. Totally1624 participants were divided and analyzed in line with the baseline and follow-up dyslipidemia status. Approximately one-third of these subjects were in the persistently healthy group (34.6%), while 39.7% and 39.6% of them were in the dyslipidemia in baseline or follow-up group and the persistently dyslipidemia group, respectively. Generalized linear model analysis showed that dyslipidemia was related to the elevated longitudinal telomere length change. According to results, dyslipidemia in the baseline or follow-up group and the persistent dyslipidemia group was the correlative factor that positively contributed to the longitudinal change in telomere length compared with the persistently healthy group. The findings further suggested that persistent dyslipidemia had a greater effect on the longitudinal change in telomere length than dyslipidemia in the baseline or follow-up group. The relative change trend of longitudinal telomere length in this study over the past 10 years is consistent with the results reported in other studies [22].

The current work mainly aimed to analyze the relation of dyslipidemia, lipid indicators and longitudinal variation in telomere length. By grouping dyslipidaemic states at baseline and follow-up and analyzing with the generalized linear models, it was discovered that the longitudinal variation in telomere length was greater and statistically significant in subjects with persistent dyslipidemia, also in females and in those aged over 60 years. This may illustrate the different results at different ages, since the older people are more likely to be affected because of the decline in all body functions. Moreover, the gender differences in body composition between females and males, and the higher percentage of body fat, explain the differences in results between males and females. In addition, it has long been recognized that females generally have longer telomere lengths than males [23, 24], and there are also studies suggesting that this difference expands throughout life. In addition, it indicates that there may be different mechanisms or influencing factors for telomere shortening between males and females [25, 26]. As a common disease in human aging, dyslipidemia is partially related to telomere length, the “marker” of aging. It is possible that the process of telomere shortening promotes the occurrence of dyslipidemia, or the occurrence of dyslipidemia accelerates telomere shortening. When comparing the correlation of alterations of TL with lipid indicator levels, it was found that HDL-C at baseline and follow-up were significantly related to alterations of telomere length. The occurrence of dyslipidemia is often accompanied by changes in some inflammatory markers, and both inflammation and oxidative stress may be correlated with telomere shortening [27]. Briefly, existing studies have not confirmed the causal relationship between the two [28, 29], which still needs to be further explored. However, the results of this study suggested an association between dyslipidemia and telomere length, especially in older people and males, which needs to be taken seriously.

In this study, 83.3% of telomere lengths were shortened, and 16.7% of telomere lengths increased after follow-up, consistent with the previous studies [30, 31]. We further explored the specific lipid indicators correlated with the longitudinal alterations of telomere length, as a result, HDL-C levels at baseline and follow-up were related to longitudinal alterations of telomere length, while lower HDL-C was related to the greater longitudinal alterations of telomere length. There are fewer studies on the longitudinal alterations of telomere length and lipid indicators. The correlation of telomere length with HDL at a cross-sectional level has been analyzed previously, although results are inconsistent [32–34]. HDL-C is significant for cardiovascular disease, while telomere length and cardiovascular disease have been a hot topic in recent years [35]. Therefore, according to the results in this study, we should raise awareness of dyslipidemia and HDL-C levels, especially in older people, because the decreases in their levels not only represent the increased longitudinal changes in telomere length, but also accelerate the risk of cardiovascular disease [36, 37]. These results have vital clinical significance for adults with dyslipidemia and abnormal lipid indicator levels.

The Q-PCR assay is a comparatively easy assay that does not need a large amount of starting DNA (approx. 50 ng). Through measuring telomere signal (T) to the reference single-copy gene signal (S), q-PCR contributes to determining T/S ratios [38]. The ratio is in proportion to the mean telomere length, which is thus used for determining relative telomere lengths [39]. While this may not be a gold standard to assess telomere length, it represents a frequently adopted method to detect more samples and can be utilized to detect less samples as well [40, 41]. However, although widely used, using the standard curves assumes perfect amplification can vary by up to 40% depending on the number of standards used, standard concentration, number of technical replicates, dilution error, and specific qPCR instrument used, especially differences in instrument [42]. As a result of these concerns, alternative, standard-free methods of estimating efficiency have been developed. T/S ratio values using the appropriate base in the formula, i.e., formula T/S=($\frac{{{E}_{T}}^{CqT}}{{{E}_{S}}^{CqS}}$)-1, where ET/S is the efficiency of exponential amplification for reactions targeting the telomere or single-copy gene respectively, and CqT/S is the cycle at which a given replicate targeting telomeric content or the single-copy gene reaches the critical threshold of fluorescence quantification [43].

### Study strengths and limitations

This study has several strengths. This is a longitudinal study including over 1600 well-characterized participants from a cohort study in the Ningxia region of northwestern China, and reflects changes in telomere length in the general rural population over nearly 10 years. Furthermore, the subjects in this study are the representative samples obtained by random sampling, which better represent the population in rural areas of Ningxia, and the obtained results can be extrapolated to a larger population. In addition, we compared the associations of alterations of telomere length with dyslipidemia and blood lipid levels. However, the long interval between telomere length measurements at the two time points (baseline and follow-up) is a limitation of this study, but a uniform laboratory test method was used to obtain telomere length and maintain consistency.

## Conclusion

In summary, after analyzing the relationship between dyslipidemia, lipid indicators and changes in telomere length, the findings suggested that changes in telomere length are correlated with dyslipidemia and its lipid indicators, especially HDL-C, during aging. Dyslipidemia and low levels of HDL-C may be associated with increased longitudinal changes in telomere length. These findings indicate the necessity of giving greater consideration to dyslipidaemia, particularly HDL-C levels, within our clinic, as dyslipidaemia has the potential to amplify longitudinal alterations in telomere length, particularly in relation to HDL-C levels.

### Electronic supplementary material

Below is the link to the electronic supplementary material.

Supplementary Material 1

## Data Availability

The datasets generated and/or analyzed during the current study are available from the corresponding author upon reasonable request.

## Abbreviations

BMI: Body mass index
DBP: Diastolic blood pressure
DNA: Deoxyribose Nucleic Acid
HC: Hip circumference
HDL-C: High-density lipoprotein cholesterol
LDL-C: Low-density lipoprotein cholesterol
LTL: Leukocyte telomere length
PCR: Polymerase Chain Reaction
RTL: Relative telomere length
SBP: Systolic blood pressure
SD: Standard deviation
T/S: Amount of telomere/amount of a single copy gene in each well
TC: Total cholesterol
TG: Triglyceride
WC: Waist circumference
