# Supplementary material for: Persistent dyslipidemia increases the longitudinal changes in telomere length
Source: Lipids Health Dis. 2023 Oct 18;22:173. doi: 10.1186/s12944-023-01938-5 (PMC10585729; doi:10.1186/s12944-023-01938-5)
Supplement: Supplementary file 1 — Supplementary Material 1 [file 12944_2023_1938_MOESM1_ESM.docx]

Supplement table 1. Comparison of baseline general conditions between the follow-up group and the lost follow-up group.

|  | Follow-up group | Lost follow-up group | *P-value* |
| --- | --- | --- | --- |
| N (%) | 2071(76.62) | 632(23.38) |  |
| $\bar{X}$±S |  |  |  |
| Age (years) | 49.30±11.69 | 45.55±13.65 | < 0.001 |
| BMI | 23.58±3.22 | 23.52±3.46 | 0.664 |
| WC (cm) | 81.03±9.27 | 80.64±10.34 | 0.396 |
| HC (cm) | 92.42±6.13 | 92.17±6.81 | 0.393 |
| SBP (mmHg) | 125.91±20.18 | 125.02±20.39 | 0.338 |
| DBP (mmHg) | 79.66±11.61 | 79.27±11.79 | 0.467 |
| FBG (mmol/L) | 5.71±1.12 | 5.73±0.98 | 0.819 |
| TC (mmol/L) | 3.95±0.83 | 3.88±0.86 | 0.062 |
| TG (mmol/L) | 1.33±0.95 | 1.40±0.94 | 0.094 |
| HDL-C (mmol/L) | 1.31±0.33 | 1.29±0.31 | 0.301 |
| LDL-C (mmol/L) | 2.05±0.68 | 1.97±0.64 | 0.009 |

Supplement table 2. Correlation between clinical characteristics and changes in telomere length.

|  |  | Baseline | |  | Follow-up | |
| --- | --- | --- | --- | --- | --- | --- |
|  |  | r | *P* -value |  | r | *P* -value |
| Age(years) |  | -0.069 | 0.005** |  | -0.039 | 0.119 |
| BMI(kg/m^2^) |  | -0.025 | 0.318 |  | 0.000 | 0.990 |
| WC (cm) |  | -0.017 | 0.484 |  | 0.002 | 0.935 |
| HC (cm) |  | -0.084 | 0.001** |  | -0.013 | 0.590 |
| SBP (mmHg) |  | -0.042 | 0.087 |  | -0.072 | 0.004** |
| DBP (mmHg) |  | -0.011 | 0.656 |  | -0.025 | 0.318 |
| TC (mmol/L) |  | -0.025 | 0.310 |  | -0.025 | 0.310 |
| TG (mmol/L) |  | -0.025 | 0.305 |  | 0.037 | 0.140 |
| HDL-C(mmol/L) |  | -0.055 | 0.026* |  | -0.053 | 0.032* |
| LDL-C (mmol/L) |  | 0.012 | 0.628 |  | 0.046 | 0.066 |
| FBG (mmol/L) |  | 0.023 | 0.377 |  | -0.012 | 0.628 |

**correlation is significant at the 0.01 level (2-tailed).

*correlation is significant at the 0.05 level (2-tailed).

Supplementary Table S3: Telomere Research Network Reporting Guidelines

| **ITEM** | **DESCRIPTION** |
| --- | --- |
| **Sample Type, Storage, Extraction, and Integrity** | |
| Sample type | DNA samples were extracted from whole blood collected via 2mL EDTA-anticoagulant tube using D3392-04 DNA Blood Mid Kit (Bao Bioengineering Co., Ltd., Japan) in baseline and follow-up. |
| Sample storage conditions, including temperature, duration, and buffer | Whole blood samples were stored at -80℃ ultra-low temperature refrigerator (Forma 88000 , Thermo, USA). Duration between sample collection and DNA extraction ranged from 1 to 2 yesrs with an average of 1.5 years. Whole blood samples were first taken from -80℃ to -20℃ and then from -20℃ to room temperature. |
| DNA extraction method | D3392-04 DNA Blood Mid Kit (Bao Bioengineering Co., Ltd., Japan) was utilized in this study with no modification from its instructions. |
| DNA storage conditions, including freeze-thaw cycles | DNA samples were stored at -80°C ultra-low temperature refrigerator (Forma 88000 , Thermo, USA). On average there were one freeze-thaw cycle for DNA samples between extraction and the qPCR assay. Samples were stored for 3.5 months between qPCR assay. |
| Method of testing DNA quality and integrity | The extracted DNA samples were tested for purity and concentration using a Biospec nano spectrophotometer, and OD260/OD180 was found to be qualified between 1.6 and 1.9. |
| Percentage of samples specifically tested for DNA quality and integrity | The extracted DNA samples were tested for purity and concentration using a Biospec nano spectrophotometer, and OD260/OD180 was found to be qualified between 1.6 and 1.9. About 86.5% DNA samples are qualified. |
| **qPCR Assay** | |
| PCR machine type | The real-time fluorescence quantitative PCR (Bio-Rad, Germany). |
| Source of master mix and reagents, and final reaction volume | The final reaction mix for the telomeric DNA contains 20ul. Namely 2*PerfectStartTMGreen qPCR SuperMix, 10 ul; 0.5 ul (10uM) forward primer, 0.5 ul (10uM) reverse primer, 9 ul DNA sample. |
| Telomere primer sequences and concentration | Primers are synthesized by Shanghai Sangon Biotech. The final concentrations of the primers upstream and downstream of the telomeres were 0.01 nmol/uL, 0.5 uL.  Forward Primer:  5＇GGTTTTTGAGGGTGAGGGTGAGGGTGAGGGTAGGGT3＇  Reverse Primer:  5＇TCCCGACTATCCCTATCCCTATCCCTATCCCTATCCCTA3＇ |
| Single copy gene name, primer sequences, and concentration | Single copy gene was *36B4* gene encoding a ribosomal phosphoprotein. Primers are synthesized by Shanghai Sangon Biotech. The final concentrations of the primers upstream and downstream of the 36B4 gene were 0.01 nmol/uL, 0.5 uL.  *36B4* Forward Primer 5＇CAGCAAGTGGGAAGGTGTAATCC3＇  *36B4* Reverse Primer 5＇CCCATTCTATCATCAACGGGTACAA3＇ |
| Full PCR program description including temperature, times, and cycle numbers | Telomeres: 95 °C – 10min; 1 cycle  95 °C – 15s followed by 54 °C for 58s; 30 cycle  36B4: 95 °C – 10min; 1 cycle  95 °C – 15s followed by 58 °C for 58s; 30 cycle |
| PCR efficiency of single copy gene and telomere primers | The relative ratio (36B4/β-globin) for all experimental DNAs in comparison with the reference DNA was ~1.0 (average = 1.0; range = 0.97 – 1.08), as expected, indicating that equal copy numbers of the 36B4 gene per cell were amplified in all DNA samples. |
| Source and concentration of standard curve | PCR reactions were followed by lysis curve analysis. The data collection was done by the supporting software of the real-time fluorescence quantitative PCR instrument. According to the principle of 2-fold exponential increase of PCR reaction products, when the amplification curve reaches the pre-set threshold in the exponential period, the software will record the Ct value of telomeres and the number of cycles of the 36B4 gene, and the relative length of DNA leukocyte telomeres can be found according to the formula T/S = [2^Ct(telomere)^ / 2^Ct(36B4)^]^-1^ = 2^-ΔCt^. |
| **Data Analysis** | |
| Number of sample replicates | Each sample was assessed for T and S on a single run with two replicates within the run. If the sample did not pass quality control criteria described below it was run a second time. |
| Level of independence of replicates | Replicates were drawn from the same DNA aliquot (i.e., the same tube). |
| Analytic method, considering replicate measurements, to determine final length | At last, the calculation of relative T/S ratio, which reflected RLTL, was made using the ΔΔCt method based on the equations below: T/S = [2^Ct(telomere)^ / 2^Ct(36B4)^]^-1^ = 2^-ΔCt^, RLTL = 2^-ΔCt^ (need checking sampling) / 2^-ΔCt^ (reference gene). |
| Method of accounting for repeated samples | samples from different time points of the same participant were different time points run as different batches. |
| ICC of study to assess reproducibility | This study is somewhat reproducible |
| Interassay and intra-assay coefficient of variation across replicate telomere measurements | N/A. No Interassay and intra-assay coefficient of variation across replicate telomere measurements were conducted. |
